# Coinfection of *Chlamydiae* and other Bacteria in Reactive Arthritis and Spondyloarthritis: Need for Future Research

**DOI:** 10.3390/microorganisms4030030

**Published:** 2016-08-24

**Authors:** Henning Zeidler, Alan P Hudson

**Affiliations:** 1 Division of Clinical Immunology and Rheumatology, Hannover Medical School, Hannover 30625, Germany; 2 Department of Immunology and Microbiology, Wayne State University School of Medicine, Detroit, MI 48201, USA; ahudson@med.wayne.edu

**Keywords:** *Chlamydia trachomatis*, *Chlamydia pneumoniae*, infection, inflammatory arthritis, undifferentiated spondyloarthritides, coinfection

## Abstract

Reactive (inflammatory) arthritis has been known for many years to follow genital infection with the intracellular bacterial pathogen *Chlamydia trachomatis* in some individuals. Recent studies from several groups have demonstrated that a related bacterium, the respiratory pathogen *Chlamydia pneumoniae*, can elicit a similar arthritis. Studies of these organisms, and of a set of gastrointestinal pathogens also associated with engendering inflammatory arthritis, have been relatively extensive. However, reports focusing on coinfections with these and/or other organisms, and the effects of such coinfections on the host immune and other systems, have been rare. In this article, we review the extant data regarding infections by multiple pathogens in the joint as they relate to engendering arthritis, and we suggest a number of research areas that must be given a high priority if we are to understand, and therefore to treat in an effective manner, such arthritides.

## 1. Introduction

Genital infection by the bacterial pathogen *Chlamydia trachomatis* (*C.tr.*) has been known for many years to function as a trigger for Reiter’s syndrome (RS) and reactive arthritis (ReA) [1,2]. Recent studies further indicate a chlamydial aetiology even for patients with spondyloarthritis (SpA) [3,4]. Epidemiologic data suggest that *Chlamydia-*induced reactive arthritis (CReA) is a more common condition than previously thought, and that clinicians often fail to recognize it [5,6]. Most importantly, a recent controlled study demonstrated that CReA can be successfully treated with combination antibiotic therapy, thereby raising the possibility of a cure [7,8]. That possibility highlights the increased significance of awareness and diagnosis of arthritis and SpA elicited by *Chlamydiae*.

It has become clear during the last two decades that two chlamydial species are responsible for causing arthritis, *C.tr.* and the related respiratory pathogen *Chlamydia (Chlamydophila) pneumoniae* (*C.pn.*), and that both elicit the disease via their persistent presence in the joint [1,2,9,10]. *C.pn.* is essentially ubiquitous in all populations so far examined, raising the possibility that coinfection involving the two chlamydial species might be significant in the aetiology of ReA and/or uSpA. This possibility was reinforced by the observation that DNA from a wide variety of bacterial species can be found in the joints of patients with arthritis [11].

Polymicrobial or coinfections are well-known and of demonstrated clinical importance in infectious diseases of the oral cavity, in otitis media, in diabetic foot wound infections, chronic infection in the cystic fibrosis lung, and in other clinical entities [12]. In general, coinfections can be concurrent, as in bacterial pneumonia with *Staphylococcus aureus* complicating flu infection, or they can be closely sequential, as with respiratory viruses plus commensal bacteria such as in otitis media caused by bacterial *Streptococcus pneumoniae* or *Haemophilus influenzae* following coronavirus, respiratory syncytial virus, or adenoviral infection [13]. Importantly, in addition to such acute coinfections, chronic infections such as those involving human immunodeficiency virus (HIV) can lead to concurrent bacterial infections with *Mycobacterium tuberculosis* and other pathogens [13].

Coinfections involving *Chlamydia trachomatis* (*C.tr.*) and *Neisseria gonorrhoeae* have been described often in screening programs and clinical settings; patients with gonorrhoeae also have been reported to have a concurrent chlamydial infection in less than 1% to a high of 70% of individuals, thus demonstrating wide variation depending on the population examined. [14,15,16,17,18,19]. Other coinfections of relevance in urogenital contexts include genital mycoplasmas and genital ureaplasmas. Coinfections with *Mycoplasma genitalium* or *Ureaplasma urealyticum* biovar 2 in men with gonococcal urethritis are associated with post-gonococcal urethritis, independent of *C.tr.* [20]. Additional studies in healthy individuals, in women in a cross-sectional sexual transmission infection (STI) screening program, and in non-gonococcal urethritis and chronic prostatitis, have reported coinfections of *C.tr.* with *M. genitalium*, *M. hominis*, *U. urealyticum* and *U. parvum* in urogenital specimens [21,22,23,24,25,26,27,28,29]. These have been implicated in sexually-transmitted urogenital diseases, although the evidence of the pathogenic role of *Ureaplasma* species is questionable given its commensal state in the urogenital flora [23,24,27,28,29].

As mentioned, compelling evidence has accumulated in recent years supporting the causative role of both *C.tr.* and *C.pn.* in ReA and spondyloarthritis (SpA) [9]. In this article, we review the evidence for coinfections involving chlamydial species reported in patients with that arthritis and SpA. We discuss the potential aethiopathogenic and clinical implications of such infections, and we address the need for future basic research and clinical studies to improve diagnosis, clinical description, and treatment.

## 2. Coinfections with *Chlamydiae* in Reactive Arthritis and Spondyloarthritis

Coinfections involving *C.tr.* and *C.pn.* were first described in synovial tissue (ST) of patients with reactive arthritis (ReA), Reiter’s syndrome (RS), and rheumatoid arthritis (RA), and later also in patients with chronic ReA and undifferentiated spondyloarthritis (uSpA) [3,5,7,8,9] Most recently, for the first time, multiple intra-articular coinfections of *Chlamydiae* with *Mycoplasma* and *Ureaplasma* were reported in patients with post-venereal ReA [30,31]. As mentioned, during the last two decades it has been established that *C.tr.* and *C.pn.* also cause SpA in at least some patients, due to their ability to persist in the joint [2,3,4]. The epidemiologic prevalence of infection with *C.pn.* is significantly higher than that for *C.tr.* overall, an observation which provided a reason for Schumacher and colleagues to assess the incidence of *C.pn.* DNA compared to that of *C.tr*. in ST and several synovial fluid (SF) samples from patients with ReA, other arthritides, and in normal joints [9]. Only 12.7% of the samples (*n* = 217) were positive for *C.pn.* compared to 28.8% for *C.tr.* Importantly, 2.4% were positive for both organisms; 5.3% of patients diagnosed with ReA; and 4.7% diagnosed with RA were PCR-positive for *C.pn.* DNA. No clear differentiating clinical or other features were identified in the patients positive for both chlamydial species, which is the reason it was not possible to decide which of the two was the causative agent for disease in these cases. Interestingly, genital infection with *C.tr.* is responsible for eliciting up to half of all cases of ReA, while pulmonary infection with *C.pn.* is responsible for less than 15% of cases. The basis for this discrepancy is unknown, but must be related to details of the genetic component of each. However, the elicitation of significant levels of synovial inflammation by either *C.tr.* or *C.pn.* does appear to be accomplished by congruent means. That is, transcription of the highly proinflammatory chlamydial hsp60 protein is upregulated during persistent synovial infection in both of these chlamydial species [32,33].

Undifferentiated SpA has been suggested to be a forme fruste of ReA, based on indirect serological evidence of preceding genitourinary or enteric infection [34]. Carter and colleagues investigated the prevalence of *C.tr.* and/or *C.pn*. DNA by PCR in ST and peripheral blood monocytes (PBMC) in patients with chronic uSpA (*n* = 26), using patients with osteoarthritis (OA) (*n* = 167) as controls [3]. Thirty-eight percent of patients with uSpA were positive in ST and 38% for *C.tr.*, 15% for *C.pn.*, and 8% for both together; OA patients were 11%, 0%, and 0.6% positive, respectively. Only 2 patients with uSpA had a history of possible *C.tr.* infection, and none had a history of *C.pn.* infection. PBMC were positive for chlamydial DNA in only 4/26 (15%) patients with uSpA (3 *C.tr*.; 1 *C.pn*.); of those, 2 were positive in ST (1 *C.tr.;* 1 *C.pn.*). Together, these data suggested that chlamydial infections, which are often occult for both organisms, are aetiologic for many patients with uSpA. Interestingly, some patients with OA, a degenerative disease without presumed infective aetiology, were found also positive for chlamydial DNA, however, less frequently than those with ReA; this observation clearly suggests that some level of subclinical, essentially invisible, background infection is present in the populations examined.

The question of the role of *Chlamydiae* as innocent bystander or causative agent in joint disease was investigated in a double-blind, placebo-controlled six-month trial with combination antibiotics (doxycycline 100 mg twice daily and rifampin 300 mg daily, azithromycin 500 mg daily × 5 days then twice weekly and rifampin 300 mg daily). In patients with chronic CReA, all were PCR-positive for *C.tr.* or *C.pn.* DNA in PBMCs and/or ST [7]. Sixty-three percent of patients undergoing active treatment were responders compared to 20% under placebo. Six (22%) patients undergoing antibiotic treatment experienced complete remission, compared to none in the placebo arm. Most interestingly, 5/6 patients who went into remission were in the azithromycin and rifampin treatment arm, suggesting this combination is most effective. In this study, coinfections of *C.tr.* plus *C.pn.* were seen in PBMC in 3 and in ST in 2 patients of the 42 included in the trial. The 2 patients positive for coinfection in PBMC and who were undergoing combination antibiotic treatment were negative after six months, in contrast to the patient under placebo who remained positive after six months. A recent case report underlines the efficacy of chlamydial coinfection in ReA. A patient (with convincingly demonstrated coinfection-positive culture for *C.tr.* and *C.pn.* in SF, culture positive bronchoalveolar lavage (BAL) for *C.pn.*, real time polymerase chain reaction (RT-PCR) positive for both *Chlamydia* spp. in SF, and RT-PCR positive in BAL for *C.pn.*) achieved complete remission with the antibiotic combination of azithromycin plus rifampicin for three months, and another two months, after discontinuing of the medications for one month to induce the persistent organisms to return to their active developmental cycle [6].

The results of the controlled trial and the case report are promising and support the causative role of *Chlamydiae* in arthritis, but those results also engender several questions: (1) Why, in the controlled trial, were about one-third of patients non-responders, and why was the rate of complete remission rather low? (2) Which is the most efficacious combination of antibiotics for treatment, and is there a need to optimize the dosing and duration of therapy? (3) Is it possible that in the controlled trial coinfections involving other bacteria associated with ReA were not identified, which may prevent the response to the treatment regimen?

The latter is an obvious possibility, given the most recent report of multiple coinfections of *Chlamydial* spp., *Mycoplasma*, and *Ureaplasma* in patients with post-venereal ReA [30]. The case study of post-venereal ReA (*n* = 22) assessed the presence of *C.tr.*, *C.pn.*, *M.hominis*, and *U. urealyticum* in samples of ST, SF, and PBMC at the time of synovectomy and after four-month antibiotic combination therapy (a combination of ciprofloxacin, tetracycline, and roxithromycin). Coinfections with two or three different bacteria were detected in 16/22 (72.7%) patients, most frequently in ST (8/17; 47.1%; *n* = 3 *C.tr.* plus *C.pn.*, *n = 4 C.tr.* plus *M. hominis*, *n* = 1 *C.tr.* plus *C.pn.* plus *M. hominis*) and PBMC (10/22; 45.5%; *n* = 6 C. tr. plus *U. urealyticum*, *n* = 1 *C.tr.* plus *C.pn.*, *n* = 1 C. tr. plus *M. hominis*, *n* = 2 *C.tr.* plus *C.pn.* plus *M. hominis*, *n* = 1 *C.tr.* plus *C.pn.* plus *U. urealyticum*) samples [30]. After synovectomy combined with antibiotic combination, *C.tr.* was found in PBMC samples from 13/22 patients. At diagnosis, 7 patients were positive for *C.pn.* and 6 for *M. hominis*. After the therapy, 4 were still positive for *C.pn.*, and one patient remained positive for *M. hominis*. Before therapy, 9 patients were positive for *U. urealyticum*, all of whom became negative after therapy. The synovectomy probably contributed notably to the remission of patients because the hypertrophic ST containing infectious agents was removed [30].

No intra-articular coinfection of *C.tr.* and *N. gonorrhoeae* has been reported to date, to the best of our knowledge, although urogenital coinfections are frequently described. However, other coinfections of *Chlamydiae* and arthritogenic bacteria are of some relevance. *Borrelia burgdorferi* is one of the most important arthritis-triggering organisms in western countries and is therefore important in the differential diagnosis of chlamydial arthritis. The simultaneous detection of DNA from *C.tr.* and *B. burgdorferi* in the SF of 6 patients with unexplained oligoarthritis was first described by Putschky and colleagues [35]. Coincidental history of tick bite and *C.tr.* positive in urogenital smears in 2/6 patients, positive serology for both bacteria in 1 patient, and positive *B. burgdorferi* serology in combination with *C.tr.* positive in urogenital smears in 1 patient, all support to some extent the suggestion that both bacteria may be causing the joint inflammation in these individual cases. Less convincing is the implicated role of coinfections involving *Yersinia enterocolitica*, *C.pn.* and *Mycoplasma pneumoniae* in *Borrelia* arthritis, which are based merely on serology and a lymphocyte transformation test [36].

Thus, evidence for coinfections of *Chlamydia* spp. with one another and with *Mycoplasma* and/or *Ureaplasma* species in joints, based on molecular genetic testing, are available for ReA, uSpA, and undifferentiated oligoarthritis from case reports and from case series studies. The pathogenic and clinical implications will be discussed in general in analogy to evidence from other established bacterial coinfections and a few relevant in vitro studies.

## 3. Aethiopathogenic and Clinical Implications of Coinfection

### 3.1. Aethopathogenesis

The traditional reductionist approach of defining details of pathogenesis by studying single bacterial infections in isolation is no longer adequate for understanding that process in polymicrobial/coinfection contexts. This is especially true in anatomic contexts which normally include a complex microbial community, such as the gastrointestinal tract and the oral cavity. The reductionist approach is also inadequate to elucidate pathogenic mechanisms in disease contexts involving complex biofilms [12]. Indeed, the presence of nonpathogenic organisms or opportunistic pathogens at low levels at an anatomic site with one or more pathogens can attenuate pathogenesis, or it can function with the pathogen(s) to increase damage. In many cases, it is simply not possible at present to define with certainty the detailed contribution of each organism (in a coinfection context) to the overall panel of pathogenic features. To cite just one set of examples of the latter from an earlier report from our group, ST and/or SF samples from more than 200 patients with a variety of arthritides were studied using a multiplex PCR system capable of identifying organisms from many genera [11]. Samples chosen for study were known to be PCR-negative for *C.tr.*, *C.pn.*, *B. burgdorferi*, and several *Mycoplasma* species, since one purpose of the study was to determine whether bacteria other than those known for eliciting joint disease could be identified in the patients with ReA, RA, OA, psoriatic arthritis, and other diseases. Of the 237 patient DNA samples, 23 (9.7%) were PCR-positive, all but 2 of which were ST. Organisms identified via DNA sequence analysis of the generated PCR products included those from the genera *Pseudomonas*, *Moraxella*, *Acinetobacter*, *Salmonella*, and others; 8/23 PCR-positive samples proved to be multiply infected, with organisms from the genera *Xanthomonas*, *Stenotrophomonas*, *Enterobacter*, and others identified in addition to the relevant index organism. We could not identify any specific aspect of synovial pathogenesis that was attributable to either of the organisms in the multiply infected samples. Here we will not address issues relating to or resulting from biofilm formation or infection of normal complex microbiological communities in human. Because only limited data regarding coinfection in ST and/or SF samples from ReA, SpA or other relevant patients are available currently, we will explore the effects of coinfection on relevant and other organisms and/or the host immune response to them, in in vitro systems or in vivo animal model systems. One set of such studies was published by our group a number of years ago. The first was intended to examine the effect of mycoplasma contamination on cultures of *C.tr.* and *C.pn.*, with the goal of clearing those cultures of the contaminating organisms [37]. Five of 9 *C.tr*. (both genital and trachoma strains) and 5/16 *C.pn.* isolates were confirmed to include mycoplasma contamination, and restriction analyses plus selective DNA sequencing of 16S rRNA produced by PCR identified *M. hominis*, *M. fermentans*, and *M. hyorhinis* in those isolates. Growth of both *C.tr.* and *C.pn.* in culture was substantially inhibited in cultures using the isolates contaminated with the various *Mycoplasma* species, compared to growth in cultures seeded with non-*Mycoplasma*-containing strains. It was not clear what effect synovial coinfection with *Chlamydiae* and *Mycoplasmae* might have on disease induction or duration, but the speculation was put forth that both might be made worse if the host immune response to *Chlamydiae* was attenuated in that situation. In a related study of *C.pn.* and *C.tr.* infection of human monocytes in culture, prostaglandin E2 (PGE_2_) production was demonstrated to be induced, although such production was not as high as that induced by treatment of the monocytes with lipopolysaccharide (LPS) from *E. coli* [38]. Interestingly, production of PGE_2_ was higher in cultured monocytes infected with *C.tr.* compared to similar cultures infected with *C.pn.*, but when cultures were infected with strains/isolates of either chlamydial species along with *M. fermentans*, PGE_2_ production was increased in a synergistic manner. Thus, coinfection of human cells in culture with either chlamydial species plus *Mycoplasmae* affects not only growth of *Chlamydiae* but also the host response to that infection.

Studies from another group investigated a relationship between coinfection with *C.tr.* and *Ureaplasma parvum*, the latter a common commensal in the human female genital system [39]. Treatment of *Chlamydia*-infected cultures of HeLa, HEp-2, or other cell types with IFN-γ results in intracellular *Chlamydiae* transiting to the persistent infection state [1,5,10,40]. Coinfection of *C.tr.* and *U. parvum* released *C.tr.* in infected, IFN-γ-treated HeLa cells from persistence; however, in the absence of IFN-γ the presence of *U. parvum* attenuated chlamydial growth. In host HeLa cells, IFN-γ inhibits tryptophan production via induction of the enzyme indoleamine 2,3-dioxygenase (IDO), and this amino acid is a requirement for intracellular growth and maturation of *C.tr.* [41]. In *U. parvum*-/IFN-γ-treated cultures of *C.tr.*-infected Hela cells, presence of the former organism either in viable or heat-killed form had no effect on IDO gene expression or enzyme production.

A recent review described studies of respiratory system coinfection with *Streptococcus pneumoniae* and other common human pathogens [42]. This is of particular interest because the organisms at issue are mucosal pathogens, as are *C.tr.* and *C.pn.*; synovial pathogenesis is a sequela of prior mucosal infection with either of the latter organisms. Coinfection with *S. pneumoniae* and *Haemophilus influenza* is common in most populations studied, particularly in children. In in vitro co-culture studies, the former has repeatedly demonstrated a clear growth and fitness advantage over the latter, but the converse is true in coinfection studies in a murine model. Investigation of the mechanism of this in vivo effect implicates a complex modulation of the host immune response, which results in a selection for virulent *S. pneumoniae* strains. Conversely, during coinfection with *S. pneumoniae* and *S. aureus*, the former strongly attenuates carriage of the latter, probably also via an immune-mediated mechanism. During coinfection with *S. pneumoniae* and influenza virus, the latter attenuates immune suppression of the bacterium, engendering a synergism in pathogenesis between the two. Thus, the interaction of multiple pathogens at the mucosal surface is a complex process, the outcome of which, in terms of pathogenesis, is not a combinatorial result of individual pathogenic mechanisms.

An issue of interest is the observation of post-gonococcal ReA suggestive of chlamydial aetiology due to urogenital coinfection of *C.tr.* and *Neisseria gonorrhoeae* but missing confirmation of the intra-articular coinfection. There are several possible explanations for that lack: (1) No targeted search in joint samples in the acute phase of gonococcal arthritis and/or post-gonococcal arthritis. Only one study searched for *Chlamydia*, *Ureaplasma*, and *Neisseria* DNA in SF from patients with inflammatory arthritis (*n* = 61), including a small number clinically associated with venereal infection (*n* = 5 gonococcal arthritis, *n* = 5 RS/ReA) with no observation of coinfection [43]. One patient with gonococcal arthritis was diagnosed because of SF-positivity by PCR for *Neisseria* DNA; proven *Chlamydia* cervicitis was negative for coinfection with *C.tr.* in the joint [43]. (2) *N. gonorrhoeae* itself may induce an aseptic ReA in susceptible patients who are treated with penicillin early and adequately, allowing the gonococci to survive in the accessory glands or the oviducts; this hypothesis was suggested on the basis of similar clinical pattern compared with RS and greater lymphocyte stimulation induced by gonococcal antigen, which was more significant in patients with post-gonorrhoeal aseptic arthritis than in healthy controls [44]. (3) The antibiotic regime against gonococci does not simultaneously eradicate coincident chlamydial infection, which allows *Chlamydia* to invade the inflamed joint by dissemination from the urogenital reservoir.

### 3.2. Diagnostic Implications

The diagnosis of ReA is most commonly based on the history of preceding infection, urogenital testing for *C.tr.*, and serology for antibodies against arthritogenic bacteria. It is rare that ST and SF samples are available for molecular testing targeting bacterial DNA in research or clinical laboratories; no commercial test kits are approved for routine application in the rheumatological setting. Thus, there is a pressing need to address the diagnostic identification of coinfections in arthritis:

(1). First, a research initiative is needed to investigate patients with undifferentiated arthritis, ReA, and undifferentiated SPA for intra-articular coinfections involving *Chlamydiae* and urogenital *Mycoplasma* and *Ureaplasma* spp.; the aim is to confirm the finding mentioned above in post-venereal ReA, which to date has been reported in only in one research unit, and to expand the investigation to the entire spectrum of arthritides which are potential consequences of coinfections.

(2). It will also be of interest to determine whether other coinfections may be found (e.g., *Chlamydia* plus enteric bacteria) with *M. pneumoniae*, *M. fermentans*, *M. salivarium* and *M. arthritides* which have been implicated as causative agents of arthritis, and whose DNA have been found by PCR in the SF of divers’ joint diseases [45,46,47,48,49] Of note, a recent study reported coinfections of *M. pneumoniae*, *M. hominis*, and *M. arthritides* in SF samples of patients with RA, using a multiplex PCR method developed for rapid and simultaneous identification of these species [50].

(3). The development of sensitive and specific multiplex molecular testing methods will be necessary to address the topics raised here, since in contrast to septic arthritis the number of non-culturable bacteria found in the joints of patients with chlamydial and other ReA is usually quite low. In our experience DNA extraction methods and PCR protocols must be adapted to the SF and ST milieu to reach adequate levels of performance [51,52,53,54]. We assume that research laboratories will develop and test protocols designed for use with joint samples for the identification of *Mycoplasma*, *Ureaplasma*, and enteric. This approach was applied to the simultaneous detection of *Mycoplasma* spp. in SF samples from patients with RA by multiplex PCR [50].

(4). Translation of research protocols and data into clinical practise requires the development of commercially available test kits adapted for SF analyses. The Anyplex II STI-7 kit (STI-7, Seegene, Eurobio)—marketed to simultaneously detect *C.tr.*, *Mycoplasma* spp., and *Ureaplasma* spp. involved in sexually transmitted infections—has identified coinfections in patients screened for genital infection and thus gives promise that the development of multiplex RT-PCR assay for the use in rheumatic patients can be realized [55]. Adaption for joint samples will be needed; our earlier observations demonstrated that commercial assays developed for urogenital samples allow the detection of *C.tr.* in clinical specimens, but they do so with a lower sensitivity than do our in-house developed *omp1*-specific PCR in concert with optimised sample preparation of SF [56].

(5). *Chlamydiae* are disseminated in monocytes/macrophages from the original site of infection by peripheral blood. They settle into the joint as shown by demonstration of chlamydial DNA in PBMC preparations from patients with ReA and undifferentiated SpA [3,57]. Hence, an important additional research topic centres on investigation of peripheral blood for detectable coinfections in patients with arthritis. The development of commercial multiplex PCR assays for peripheral blood testing is of great importance for rapid diagnosis, and to overcome the limited availability of joint samples in every day clinical practise.

The comprehensive research program outlined here is a prerequisite to understand the clinical implication of chlamydial coinfections in arthritis and SpA, and to develop therapeutic strategies directed against multiple persistently-infecting organisms.

### 3.3. Clinical Implication

The disease course of ReA is self-limiting, remitting, or chronic progressive. A few factors are known to be associated with the progression from acute ReA to chronic SpA: the presence of HLA-B27, a positive family history for ankylosing spondylitis or SpA, and the presence of chronic lesions in the gut [58]. One can envision several scenarios explaining how coinfections might impact CReA:

(1). Coinfections may induce more severe inflammation in the arthritic joint during the acute phase of disease, and/or.

(2). They may prolong the self-limiting phase of the disease, and/or.

(3). They may cause relapse of the disease, and/or.

(4). They may support the chronic disease course because one or several of the coinfecting bacteria are not eliminated.

In vitro studies have shown that coinfections of *M. fermentans* with *C.tr.* and *C.pn.* suppress chlamydial growth, which in turn may induce persistent infection resulting in additive stimulation of the PGE_2_ production in human monocytes [37,38]. Elicitation of chlamydial persistence by either species in the synovium via whatever means virtually guarantees significant and sustained levels of local inflammation. As given in detail in other publications, persistently infecting *Chlamydiae* display a number of unusual morphological and molecular genetic characteristics, all of which contribute to production of inflammation in the host. Morphology for persistent *C.tr.* and *C.pn*. both is highly aberrant, a reflection of the severe attenuation of production of the immunodominant, shape-determining major outer membrane protein (omp-1) of these organisms. This selective transcriptional attenuation is just one facet of a major adjustment in the overall panel of chlamydial gene expression which characterises persistence. As mentioned above, probably the most critical adjustment relevant to inflammation is the strong upregulation of expression of the bacterial heat shock protein-encoding (hsp) genes, the products of which are recognized by the host as powerfully proinflammatory [32,33]. Other molecular genetic aspects of persistence also contribute variously to induction and maintenance of inflammation [10,59,60,61]. All these effects are factors that may cause more severe, prolonged, and even chronic disease. Further and as described above, coinfection with *C.tr.* and *U. parvum* in an in vitro HeLa cell system treated with IFN-γ promotes maturation of *C.tr.* from reticulate bodies to elementary bodies independent of IDO expression; clearly this suggests a novel survival strategy of *C.tr.* against IFN-γ exposure, which is of course a critical host defence factor for eliminating *Chlamydiae* [39].

### 3.4. Treatment

As already mentioned, the question arises as to why, in the double-blind, placebo-controlled six-month trial with combination antibiotics (doxycycline 100 mg twice daily and rifampin 300 mg daily, azithromycin 500 mg daily × 5 days then twice weekly and rifampin 300 mg daily) in patients with chronic CReA [7], about one-third of patients were non-responders, and why the rate of complete remission was rather low. Supposing that coinfections involving other bacteria associated with ReA were not identified, in that case, the effectiveness of the antibiotics against the candidate bacteria must be questioned. For example, doxycycline effectively has a low eradication rate for *M. genitalium,* and the eradication rate with azithromycin is decreased significantly due to rapid emergence of resistance [62]. A 5 day azithromycin treatment regimen—500 mg on day 1 and 250 mg on the following 4 days—is needed to effectively eradicate urogenital coinfections of *C.tr.* and *M. genitalium*. This may explain why the combination of azithromycin and rifampin was most effective in inducing remission in chronic CReA, although chlamydial infection alone can be effectively eliminated by the combination of azithromycin and rifampin in vitro in HEp-2 cells [63]. In summary, further studies are necessary to identify the most effective antimicrobial combination therapy, including that for coinfections, to cure chlamydial arthritis.

## 4. Conclusions

Several recent reports of coinfections cited herein involving *Chlamydiae*, *M. hominis*, and *U. urealyticum* in patients with post-venereal ReA indicate a clear and critical unmet need for future research to understand the nature and consequences of coinfections for diagnostics, clinical course, and treatment of chlamydial arthritis and SpA. In particular, more holistic data collection—including the large spectrum of the potential urogenital, respiratory, and even enteric pathogen candidates for coinfections—would help to advance understanding of the role of bacteria in arthritis and SpA. Improved knowledge is needed regarding the risk factors for coinfections, the clinical circumstances in which *Chlamydiae* interact with other pathogens, and the mechanisms behind such pathogen–pathogen interactions, including experimental studies.
